# DNA Catalysis: The Chemical Repertoire of DNAzymes

**DOI:** 10.3390/molecules201119730

**Published:** 2015-11-20

**Authors:** Marcel Hollenstein

**Affiliations:** Department of Chemistry and Biochemistry, University of Bern, Freiestrasse 3, CH-3012 Bern, Switzerland; hollenstein@dcb.unibe.ch; Tel.: 41-31-631-4372

**Keywords:** SELEX, DNAzymes, chemically modified nucleic acids, functional nucleic acids, biosensors, therapeutic nucleic acids

## Abstract

Deoxyribozymes or DNAzymes are single-stranded catalytic DNA molecules that are obtained by combinatorial *in vitro* selection methods. Initially conceived to function as gene silencing agents, the scope of DNAzymes has rapidly expanded into diverse fields, including biosensing, diagnostics, logic gate operations, and the development of novel synthetic and biological tools. In this review, an overview of all the different chemical reactions catalyzed by DNAzymes is given with an emphasis on RNA cleavage and the use of non-nucleosidic substrates. The use of modified nucleoside triphosphates (dN*TPs) to expand the chemical space to be explored in selection experiments and ultimately to generate DNAzymes with an expanded chemical repertoire is also highlighted.

## 1. Introduction

For a long time DNA was thought to be the repository of genetic information, serving solely as the blueprint of life. However, due its inherent structural properties, DNA and chemically modified analogs have advanced as warhorses in multiple applications, including the construction of nanomaterials [1,2], the development of therapeutic agents [3,4], the creation of diagnostic tools and sensors [5,6,7], and the design of logic gates and computation circuits [8,9,10,11]. Paralleling these developments, the chemical functions of DNA have been explored beyond its double helical nature and culminated in the isolation of potent aptamers, *i.e.*, single-stranded oligonucleotides that bind to specific targets with high affinity and selectivity and that are often considered as being the chemical equivalent of antibodies [12,13,14,15]. Indeed, the advent of the Systematic Evolution of Ligands by EXponential enrichment (SELEX) technique and related combinatorial methods of *in vitro* selection [16], has propelled the development of aptamers for a broad range of targets.

Interestingly, the folding properties and inherent chirality of the double helical DNA have also been exploited to develop DNA-based catalysts. Catalytic DNA consists either of: (1) supramolecular hybrids made of metal complexes and double-helical DNA molecules [17,18,19] or (2) of single-stranded DNA sequences (coined DNA enzymes, deoxyribozymes, or DNAzymes) obtained by *in vitro* selection [20,21,22].

While DNAzymes were originally conceived as potential gene silencing agents due to their capacity to specifically and selectively cleave stretches of mRNA targets [23,24,25], numerous DNAzymes catalyzing a broad array of chemical transformations have been isolated, thus expanding their scope and instating them in the role as true biocatalysts along with ribozymes and proteinaceous enzymes. Therefore, this review article provides an overview of the diversity of the chemical repertoire of DNAzymes and will discuss all the chemical reactions catalyzed by these functional nucleic acids. Moreover, strategies for improving both the catalytic efficiency and the scope of these biomolecular catalysts will be highlighted, with a strong emphasis on the use of modified nucleoside triphosphates (dN*TPs) in selection experiments.

## 2. DNAzymes as Bond Cleaving Catalysts

**Figure 1:**
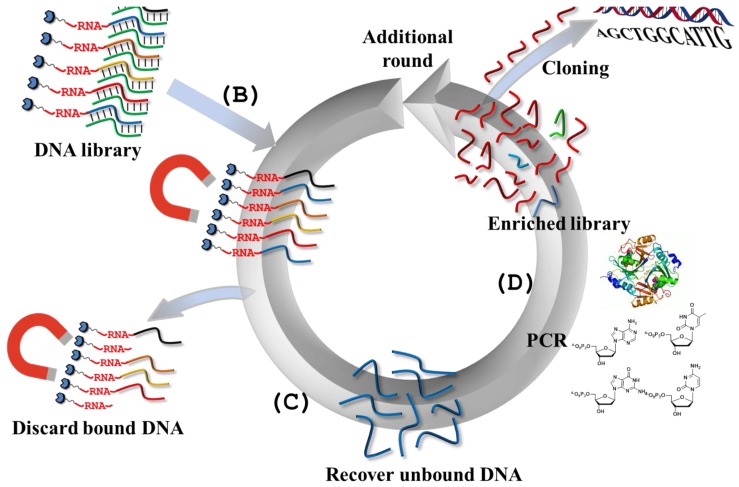
Overview of the selection scheme for the generation of RNA-cleaving DNAzymes: (**A**) oligonucleotides containing fixed-sequence regions (shown in blue) for the docking of the PCR primers and a central randomized region (in green) obtained by solid-phase synthesis, constitute the starting point for the generation of a randomized library either by PCR or by primer extension reaction. The primers often have biotin residues appended at their 5′-ends to enable immobilization of the oligonucleotide population and contain the RNA substrate; (**B**) the library is immobilized on a solid support (e.g., streptavidin-coated magnetic particles) and if necessary, the template strand is stripped off by a hydroxide treatment; (**C**) the immobilized population of oligonucleotides is subjected to the reaction conditions, and only the catalytically active species will be separated from the solid support and eluted; (**D**) the eluted sequences are PCR amplified and used in a subsequent round of selection. Adapted from reference [15].

Shortly after the advent of SELEX for the generation of aptamers [26,27,28], this method of molecular Darwinian evolution was adapted to isolate the first DNAzyme, designed to act as an artificial ribonuclease [20]. This method is highlighted in Figure 1 and only deviates slightly from the original protocol devised for the isolation of aptamers. Briefly, the selection process starts with the generation of an initial population of oligonucleotides bearing fixed-sequence regions at both ends (for the docking of PCR primers) and a central randomized region, typically N_20_ or N_40_ (Figure 1A). This library—generally containing up to 10^16^ molecules and generated by PCR or primer extension reactions with primers containing the scissile units (e.g., RNA nucleotides)—is then immobilized on a solid support and incubated under the appropriate reaction conditions (Figure 1B,C). Only the catalytically proficient species will be capable of cleaving their own release from the solid support (Figure 1C) and will be PCR amplified (Figure 1D) and used in subsequent rounds of selection [29]. The reaction conditions often involve diverse metal cations including Mg^2+^ [30,31], Pb^2+^ [20], Cd^2+^ [32], Ln^3+^ [33,34,35], UO_2_^2+^ [36] or small organic molecules such as histidine [37], which serve as cofactors for the functionality depleted nucleic acid catalysts. The choice of the adequate reaction buffer is of paramount importance and has a strong influence on the outcome of the selection experiment. Recently, Lu and coworkers used a minimal buffer containing either 135 or 400 mM Na^+^, 10 mM citrate, and 1 mM EDTA to isolate an RNA-cleaving DNAzyme that was strictly and solely dependent on the presence of Na^+^ [38]. Besides the buffer composition, multiple parameters can be used to alter the stringency of the selection. For instance, an increase in stringency can involve a reduction of either the reaction time or the concentration of the cofactor, a variation of the temperature and ionic strength, an alteration of the amplification or primer extension protocols [29,39,40,41,42], or the inclusion of a splint ligation step [43] or blocking oligonucleotides [32]. The choice of the length of the random section for the generation of the initial population is another experimental variable that can have a profound impact on the outcome of selection experiments and no defined and general rules have yet been established [29,44]. For instance, catalytically more efficient DNA-cleaving DNAzymes were isolated when shorter randomized domains (N_20_ or N_30_) were used in selection experiments than longer stretches (N_50_ or N_60_), while the opposite trend was observed when the initial populations were challenged to catalyze the tyrosine-RNA nucleopeptide linkage formation [44].

**Figure 2:**
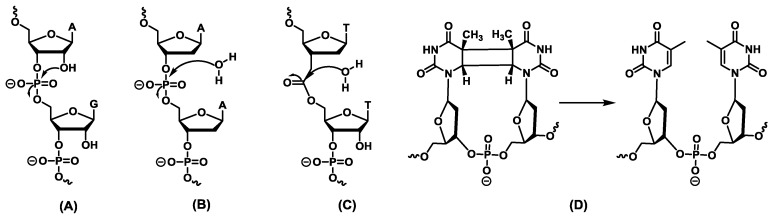
Summary of the different reactions catalyzed by bond-cleaving DNAzymes: (**A**) cleavage of ribophosphodiester linkages; (**B**) scission of deoxyribophosphodiester bonds [48,49]; (**C**) hydrolysis of ester units [50]; (**D**) photoreactivation of cyclobutane thymine dimers [51].

Application of *in vitro* selection has led to the identification of numerous DNAzymes catalyzing the scission of ribophosphodiester linkages (Figure 2A). As a matter of fact, a vast majority of the selected DNAzymes catalyze the hydrolysis of RNA substrates due to the ease of selection and the potential (*in vivo*) applications [45]. However, lured by the catalytic prowess of RNA-cleaving DNAzymes and by the inherent properties of DNA, a rich field of DNAzymes capable of cleaving various bonds has been developed (Figure 2) [46,47]. Indeed, DNAzymes capable of hydrolyzing the phosphodiester linkages of DNA (Figure 2B), ester and anilide bonds (Figure 2C), and even a DNAzyme capable of repairing thymine dimers (Figure 2D) have been isolated. The following section describes all the different DNAzymes capable of catalyzing bond-scission reactions.

### 2.1. RNA-Cleaving DNAzymes

**Figure 3:**
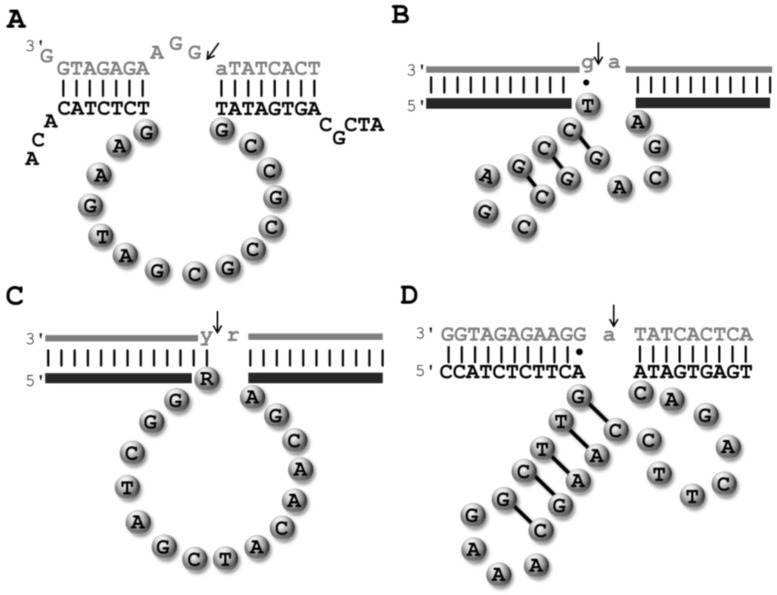
Putative schematic representations of the putative secondary structures of RNA-cleaving DNAzymes: (**A**) the first RNA-cleaving Pb^2+^-dependent DNAzyme to have been isolated by *in vitro* selection [20]; the all-RNA cleaving DNAzymes 8-17 (**B**) and 10-23 (**C**) [31]; and (**D**) the UO_2_^2+^-dependent RNA cleaving DNAzyme E_Hg_0T [59]. The substrates are shown in grey and thin lines and lower case letters represent RNA units, while thick lines and upper case letters represent stretches of DNA nucleotides. The cleavage sites are indicated by an arrow.

As aforementioned, the first DNAzyme was isolated by *in vitro* selection by Breaker and Joyce in 1994 (Figure 3A) [20]. This early selection experiment involved a substrate containing a single embedded ribo(adenosine) nucleotide and the reaction buffer contained 1 mM Pb^2+^, which was deemed to serve as the divalent metal cofactor and promote catalysis by analogy to what had been observed in the case of some ribozymes [52,53]. Starting with an initial population consisting of ~10^14^ molecules with an N_50_ randomized stretch, five rounds of *in vitro* selection led to the isolation of a Pb^2+^-dependent intramolecular (*cis*) catalyst. This DNAzyme promoted the hydrolysis of the scissile linkage with a first-order rate constant (*k*_obs_) of 1.4 min^−1^ [20], which represents an impressive ~10^7^-fold rate-enhancement compared to the background cleavage of RNA [54]. This *cis*-cleaving leadzyme was converted into an intermolecular (*trans*) catalyst (Figure 3A) that could cleave the substrate with a catalytic efficiency (*k*_cat_/*K*_m_) of 5 × 10^7^ min^−1^·M^−1^. Following this initial discovery, other DNA-based ribophosphodiesterases including Mg^2+^ and Ca^2+^-dependent DNAzymes were isolated by *in vitro* selection experiments [30,55]. Both these DNA metalloenzymes cleaved substrates containing a single ribo(adenosine) nucleotide, both in *cis* and *trans*, with catalytic efficiencies lying in the range of 10^3^–10^4^ min^−1^·M^−1^. Subsequently, two of the most proficient and complete DNAzymes, coined Dz8-17 (Figure 3B) and Dz10-23 (Figure 3C), were isolated by Santoro and Joyce [31]. These Mg^2+^-dependent DNAzymes can cleave a broad variety of all-RNA substrates (Figure 3) with efficiencies approaching kinetic perfection (~10^9^ min^−1^·M^−1^) at high Mg^2+^ concentrations (~100 mM) [31,56]. While Dz10-23 was shown to be rather tolerant to the nature of the substrate, cleaving all purine-pyrimidine dinucleotide junctions (R·Y in Figure 3C with R = A or G; Y = U or C) [31,56], Dz8-17 was initially thought to be more demanding in terms of sequence requirements of the substrate, cleaving only a G·A dinucleotide junction (Figure 3B). However, reselection of Dz8-17 variants [57] and mutation experiments [58] later revealed that Dz8-17 can be coerced into cleaving all 16 dinucleotide junctions, albeit with *k*_obs_ values spanning over five orders of magnitude.

The catalytic core of Dz8-17 consists of an important G·T wobble pair [31,60], a three-nucleotide long stem maintained by Watson-Crick pairs, an AGC trinucleotide loop, and four unpaired residues. On the other hand, the 15-nt long catalytic domain of Dz10-23 seems to be devoid of any salient structural feature and consists solely of unpaired nucleotides. Both DNAzymes share common features, including four highly-conserved residues in the catalytic core, which hints at the possibility that Dz10-23 is a structural variant of Dz8-17 [61].

Surprisingly, the Dz8-17 motif has poisoned numerous independent *in vitro* selection experiments designed to improve and extend the scope of RNA-cleaving DNAzymes [42,55,60,62,63,64,65]. The resurgence of a small catalytic motif in selection experiments, the so-called tyranny of the small motif [66], may be ascribed to the much higher statistical probability of the presence of multiple copies of a smaller sequence motif than that of comparatively larger or more complex motifs. Other factors may also contribute to the recurrence of small motifs including the capacity of smaller motifs to adopt secondary and tertiary structures, the choice of the conditions chosen in the *in vitro* selection experiment, and the ease of replication during PCR or primer extension reactions (especially in the context of modified dN*TPs) [66,67,68].

**Figure 4:**
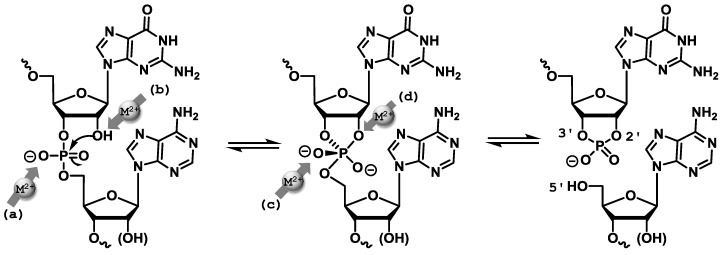
RNA-cleavage reaction (shown for Dz8-17) leading to the 2′,3′-cyclic phosphate and the 5′-OH products and plausible sites of interaction of the M^2+^ cofactors (directly or via an activated water molecule):(**a**) negative charge stabilization of a non-bridging oxygen atom or general acid catalysis; (**b**) activation of the nucleophilic hydroxyl unit by deprotonation or coordination; (**c**) stabilization of the build-up of negative charge on the oxygen atom of the leaving group; (**d**) general base catalysis [72,82,84,85]. Scheme adapted from reference [82].

In the absence of a crystal structure of an active DNAzyme [69], insight into the folding characteristics and the catalytic mechanism have been gathered by FRET analyses [70,71,72,73,74], mutagenesis and deletion experiments [61,75,76,77,78], and biochemical characterization [56,79]. In the case of Dz8-17, the induced folding preceding the catalytic activity strongly depends on the nature of the metal cation involved. Indeed, in the presence of Mg^2+^ and Zn^2+^, the metal cation induces a global folding of the enzyme which is then activated for catalysis, thus following an induced fit mechanism [71]. On the other hand, in the presence of Pb^2+^, no global folding is observed and catalysis is initiated by the binding of the divalent metal cation to the binding pocket, thus following a lock-and-key type of mechanism [71,72]. Conversely, less is known on the folding of Dz10-23 by the impulse of metal cofactors, but it appears to be similar to that of Dz8-17 triggered by Mg^2+^ and Zn^2+^ [74,80]. Indeed, the folding and catalytic activity seem to be intertwined and Dz10-23 adopts a compact, triangular pyramidal structure upon addition of Mg^2+^ [81]. At low divalent metal concentrations (~0.5 mM), the induced folding is not sufficient to promote efficient catalysis, which only occurs at higher concentrations (~5 mM) when the DNAzyme is folded in its compact structure and the flanking arms simultaneously adopt the appropriate orientation for binding to the substrate [80]. Therefore, it is believed that RNA-cleavage mediated by both Dz8-17 and Dz10-23 follows a mechanism that is similar to that of ribozymes such as the hammerhead ribozyme (Figure 4) [80,82]. Briefly, folding of the DNAzyme scaffold triggered by the presence of the divalent metal cofactors allows an adequate positioning of the substrate and the functionalities involved in the catalytic step. In addition, the M^2+^ cofactor will also activate the nucleophilic 2′-hydroxyl unit flanking the scissile bond by deprotonation and/or coordination [72]. Finally, the S_N_2-like nucleophilic attack on the phosphorous center leads to a pentacoordinate intermediate, which breaks down into the 2′,3′-cyclic phosphate and the 5′-OH products. The 2′,3′-cyclic phosphate product is further hydrolyzed to a 2′- or 3′-phosphate only in the case when Dz8-17 is incubated with Pb^2+^ [83].

The versatility and catalytic prowess of Dz8-17 and Dz10-23 have propelled these biocatalysts to the forefront of numerous applications, including their use as therapeutic nucleic acids, biosensors, and logic gate and circuits [8]. Initially, Dz8-17 and Dz10-23 were conceived as gene silencing agents and consequently numerous *in vivo* and *in vitro* assays have been conducted in order to evaluate their capacity at operating as gene regulating devices [86,87,88,89,90,91,92,93,94]. These promising results along with continuous research efforts to develop *in vivo* and *in vitro* experiments have culminated in the application of two variants of Dz10-23 in clinical trials [95,96,97]: (i) Dz13 [98] was designed so as to specifically cleave c-Jun mRNA in order to inhibit the expression of the c-Jun protein which is expressed in human atherosclerotic lesions. c-Jun is also an archetypical member of the AP-1 (activator protein 1) protein family, which has been shown to be implicated in tumorigenesis, particularly in the formation of skin tumors [99]. The gene-silencing capacity of Dz13 was exploited to treat basal-cell and squamous-cell carcinomas in different mammals including rodents and monkeys [100]. This animal study was instrumental for the development of the first-in-human phase I clinical study of a DNAzyme where Dz13 was administered by intratumoral injection to nine patients suffering from basal-cell carcinoma [96]. The level of c-Jun expression was reduced in all patients and three out of nine patients showed a significant decrease in tumour depth; (ii) a more recent phase IIa clinical trial made use of a variant of Dz10-23 coined hgd40 to treat patients suffering from allergen-induced asthma [97]. The use of a therapeutic oligonucleotide for the treatment of airway diseases might seem slightly counterintuitive, but the downregulation of the GATA-3 transcription factor represents an alluring strategy for directly interfering with the disease. Indeed, GATA-3 is the master transcription factor for the differentiation of type T-helper cells type 0 (Th0) to Th2 cells following allergen exposure which is a key step in allergic bronchial asthma since the Th2 cells mediate the production of cytokines such as interleukines IL-4, -5, and -13 which then trigger the immune response [101,102]. The hgd40 DNAzyme was obtained after an *in vitro* screening of 70 different species containing the catalytic motif of Dz10-23 and randomized binding arms in order to ensure selective recognition of the mRNA target. This DNAzyme was then shown in an *in vivo* preclinical trial to selectively hydrolyze the GATA-3 mRNA *in vitro* and to inhibit the inflammation and mucus production in mice [103]. Also, the administration of fluorescently- and radioactively-labelled hgd40 was utilized to investigate the biodistribution of the DNAzyme in mice, rats, and dogs [104]. Following these preclinical studies, treatment of patients with hgd40 (10 mg per once-daily inhalation) led to a reduction of the early and late asthmatic responses in 11% and 34% of the cases, respectively [97]. Thus, DNAzyme hgd40 presents very favorable assets for the treatment of asthma, but unanswered questions such as the advantage over small molecules still need to be addressed in future clinical phase II and III trials.

Despite these favorable results and developments, new methods for the increase of the cellular delivery and serum resistance of therapeutic oligonucleotides are needed. In this context, it was shown that the conjugation of antisense oligonucleotides to gold nanoparticles (NPs) was a valuable strategy since the resulting complexes displayed a higher affinity for complementary sequences than the unmodified counterparts, were more amenable to cells, and were capable of efficient gene knockdown [105]. Recently, Dz10-23 was conjugated to NPs and after fine-tuning of the molecular density on the particles and assessment of the length of the connecting linker, optimal conditions were found that support catalysis of these constructs [106]. Importantly, these constructs were then demonstrated to substantially reduce the expression of the growth differentiation factor 15 (GDF15) underscoring the usefulness of this approach.

The *modus operandi* of DNAzyme-mediated gene silencing is still matter of debate: numerous reports suggest that the active DNAzyme cleaves the relevant mRNA target at multiple locations causing a disruption of the translation (specific effects) [92,103,106,107], while other hint at non-specific effects where the DNAzymes prevent gene expression by creating a steric blockade of the mRNA or elicit RNase H, much like therapeutic antisense oligonucleotides [25,80,108]. The distinction between both mechanisms is rather difficult since both require binding to the mRNA target and might depend on the nature and the three-dimensional folding of the mRNA [109].

A multitude of different RNA-cleaving DNAzymes has been crafted with the aim of serving as biosensors, mainly for the detection of metal contaminants. For instance, a UO_2_^2+^-dependent DNAzyme that allows for the detection of only trace amounts of the uranyl contaminant [36], was additionally converted to a highly efficient sensor for the Hg^2+^-pollutant (Figure 3D) [59]. However, this very vast and interesting research avenue has been addressed recently by others and is beyond the scope of this review [47,110,111,112].

### 2.2. DNA-Cleaving DNAzymes

The incentive for the development of DNA-cleaving DNAzymes was spurred by: (i) the need for artificial DNA nucleases that could act as restriction enzymes, which would represent a very valuable addition to the armamentarium of tools available in microbiology and drug discovery [113,114]; (ii) precedent in RNA with the *in vitro* selection of group I intron ribozymes capable of hydrolyzing ssDNAs [115,116,117]; (iii) the possibility of expanding the catalytic repertoire of DNAzymes towards more arduous reactions (t_1/2_ for the uncatalyzed hydrolysis of RNA is ~4 [118] to 10 [54] years compared to ~140,000 [118] to 30 million [119] years for DNA); (iv) the question of the hypothetical presence of naturally occurring DNAzymes [49].

Initial selection experiments resulted in the isolation of catalytic DNAs that promoted the scission of DNA sequences either via an oxidative mechanism (with Cu^2+^ in the presence or absence of ascorbate as cofactors) [120] or by depurination of a deoxyguanosine nucleotide followed by β-elimination at the resulting abasic site [121], but not through the direct hydrolysis of the phosphodiester bonds. More recently, the base-excision capacity of catalytic DNAs was exploited in an *in vitro* selection experiment to generate a Cu^2+^/Mn^2+^-dependent DNAzyme that promotes the selective oxidative excision of thymine nucleotides (rate for *trans*-cleavage: *k*_obs_ = 2.3 × 10^−3^ min^−1^) and thus could be used as a tool for the replacement of single-nucleotide polymorphisms (SNPs) [122]. The cleavage mechanism seems to involve a reaction with molecular oxygen or hydrogen peroxide along with the reduction of Cu(II) to Cu(I) which is of importance for the catalytic activity [122,123].

**Figure 5:**
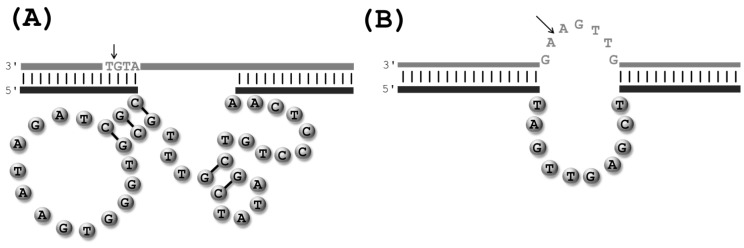
Sequences and hypothetical 2D structures of the DNA-cleaving DNAzymes 10MD5 [48] (**A**) and I-R3 [49] (**B**).

The first DNAzyme with DNase activity was discovered serendipitously by Silverman *et al.* who set out to explore the possibility of DNA-mediated hydrolysis of peptide bonds [48]. The substrate employed in this selection experiment consisted of a DNA-tripeptide chimera and the resulting catalytically active species all hydrolyzed the more robust phosphodiester bonds at various locations, rather than any of the more labile peptide linkages (t_1/2_ for the uncatalyzed hydrolysis of amide bonds is ~500 years [124]). The most active DNAzyme, 10MD5 (Figure 5A), cleaved at the ATG^T site in the presence of both the chimeric and an all-DNA substrate with similar high rates (*k*_obs_ = 0.033 and 0.045 min^−1^, respectively) and depended on the simultaneous presence of both Mn^2+^ and Zn^2+^ cofactors for activity. DNAzyme 10MD5 truly adopts a hydrolytic mechanism because when the reaction was carried out in ^18^OH_2_, incorporation of ^18^O in the phosphate unit of the 5′-product was observed, as expected for P-O bond hydrolysis [48].

Despite the remarkable catalytic traits, Dz10MD5 is intolerant to even minute changes in the pH of the reaction buffer (a delta pH of 0.2 led to a > 10-fold depletion in rate) and displays a rather poor substrate tolerance (only substrates containing the ATG^T cleavage site are recognized) and a strong necessity to bind the substrate via Watson-Crick base-pairing. Consequently, additional selection experiments were designed to improve on these negative features. Firstly, a partially randomized catalytic core of 10MD5 along with a systematic variation of the pH of the reaction buffer composed the basis of a reselection experiment that led to the identification of Dz9NL27 [125]. This DNAzyme was capable of cleaving an all-DNA substrate with appreciable rate constants (*k*_obs_ ~1.5 × 10^−3^ to 0.02 min^−1^) over a substantial pH range (from 7.2 to 7.7), albeit at the expense of a lower site specificity (miscleavage was observed). Secondly, *in vitro* selection experiments were devised to investigate on the required length of unpaired nucleotides in the substrates and subsequently to increase the sequence tolerance of DNA-hydrolyzing DNAzymes [43]. These experiments showed that substrates containing only a single unpaired nucleotide enabled a strong catalytic activity (e.g., one DNAzyme, Dz13PD1, displayed an impressive first-order rate constant of 1.2 min^−1^), while longer unpaired sequences led to sluggish catalysts and in the absence of unpaired nucleotides the catalytic species are coerced to adopt an oxidative mechanism. Also, inclusion of an additional splint ligation in the selection protocol as well as exploration of all possible combinations of XG unpaired dinucleotides in the substrate, led to the identification of numerous potent DNAzymes with a large sequence tolerance and that obligatorily proceeded via a hydrolytic mechanism [43]. Finally, the cofactor dependence could be reduced to the lone Zn^2+^ cation by a double nucleotide mutation [126] or converted to lanthanide cations Ln^3+^ (particularly Ce^3+^) by *in vitro* selection [127].

An interesting approach was adopted by Breaker *et al.* to isolate new classes of DNA-cleaving DNAzymes [49]: a circular DNA library consisting of two N_50_ randomized regions was constructed by using the ATP-dependent ligase CircLigase™ which mediates the circularization reaction of 5′-phosphorylated ssDNAs [128,129]. After incubation of the circular library in a buffer containing Zn^2+^ as a cofactor, the products stemming from the hydrolytic reaction are subjected to a second CircLigase-mediated ligation prior to PCR amplification. The ingenuity of this methodology resides in the fact that cleavage can occur at any location of the construct, even in the randomized regions, without loss of any genetic information. Simultaneously, this system also avoids the occurrence of alternate mechanisms such as depurination or oxidative pathways. After 14 rounds of selection, two classes of DNAzymes (coined I and II), differing in size and structural features, were identified and one particular representative of class I, I-R3 (Figure 5B), displayed an impressive rate constant (*k*_obs_ = 1.0 min^−1^) for the Zn^2+^-dependent hydrolysis of DNA at one specific location (*i.e.*, the A-A dinucleotide stretch indicated by the arrow in Figure 5B). Like Dz10MD5 (*vide supra*), I-R3 was very sensitive to variations in pH (optimal activity at pH 7.0) and heavily dependent on the presence of the Zn^2+^-cofactor, but on the other hand displayed a rather large sequence tolerance at the cleavage site [49]. Interestingly, I-R3 could be converted into a smaller species capable of multiple turnover which was recruited to cleave the single-stranded genome of bacteriophage M13. Lastly, the high activity and the relatively small size of the isolated DNAzymes raised the question of the existence of similar sequences in natural genomes. Even though several analogous sequences were identified using bioinformatics algorithms, none of these sequences showed any catalytic activity under cellular Zn^2+^ concentrations (~50 μM).

### 2.3. Other Bond Cleaving DNAzymes

Besides DNA catalysts cleaving P-O bonds of RNA and DNA substrates, examples of bond-cleaving DNAzymes are relatively scarce. However, notable examples include DNA-mimics of enzymes such as esterases (Figure 2C) [50], CPD-photolyases (Figure 2D), and phosphatases [51].

DNA photolyases repair DNA lesions caused by exposure to UV-radiation. The most common type of DNA damage is the formation of cyclobutane pyrimidine dimers (CPD) by a [2+2] cycloaddition and CPD-photolyases use various catalytic cofactors such as reduced flavin (FADH^−^) and light as a source of energy to reverse this reaction [130]. Sen and coworkers set out to isolate DNA enzymes that could emulate photolyases [51]. To this end, a substrate containing a thymine-thymine photodimer was synthesized and served as the primer for the generation of a randomized library. After a negative selection step which involved UV-irradiation in the absence of a cofactor, the population of DNA molecules was incubated with serotonin and subjected to UV-light irradiation (>300 nm). Sequences that were repaired in the process could be isolated by gel electrophoresis due to their lower mobility. Surprisingly, robust self-repairing was observed in the negative selection and cloning and sequencing of the populations of the 20th selection round revealed that the predominant species emanating from both selection steps were fundamentally different in terms of sequence composition. DNAzyme UV1A, which was identified from the negative selection pool, was shortened by removal of a constant region and the resulting DNAzyme, UV1C, displayed a high catalytic efficiency (*k*_cat_/*K*_m_ = 7.8 × 10^6^ min^−1^·M^−1^) for the self-repair reaction, which represents a stunning >10^4^-fold rate enhancement compared to the uncatalyzed reaction. In terms of mechanism, the formation of a G-quadruplex like structure was thought to act as the light-harvesting photoantenna involved in the efficient transmission of light to the damaged substrate, in analogy to the mechanism adopted by CPG-photolyases [131]. In a different report, single substitutions of guanine nucleotides with the fluorescent analog 6-methylisoxanthopterin (6MI) in the scaffold of DNAzyme UV1C resulted in a catalytic species that functioned at less damaging higher wavelengths (345 nm) [132]. Indeed, when guanine 23, which is believed not to be involved in the G-quadruplex structure, was substituted with 6MI (or other chromophores), the catalytic efficiency at 345 nm increased significantly compared to other mutants or the wild-type UV1C (*k*_obs_ = 0.32 min^−1^ *vs.* ~0.005 min^−1^).

More recently, substrates containing esters, aliphatic and aromatic amides, and a tripeptide were subjected to *in vitro* selection conditions to evolve DNAzymes capable of hydrolyzing these different bonds [50]. Of these selection experiments, DNAzymes that cleaved ester linkages (with a maximal rate of *k*_obs_ = 0.05 min^−1^) as well as anilides (with a rate constant for single-turnover of *k*_obs_ = 3.5 × 10^−3^ min^−1^) were generated. Surprisingly, no DNAzyme capable of cleaving aliphatic amide bonds was isolated. This observation underscores the resilience of DNAzymes to hydrolyze peptide bonds despite the relative lability of these linkages compared to, for instance, the sturdy phosphodiester bond of DNA [48]. A similar observation had been made in the case of ribozymes: a selection experiment designed to identify catalysts capable of hydrolyzing an embedded 3′-NH–C(O)–CH_2_-5′ linkage [133], resulted in a ribozyme that cleaved the DNA phosphodiester bond located 5′-upstream of the amide bond instead of the intended target [134]. This reluctance of nucleic acid-based catalysts to hydrolyze peptide bonds is often ascribed to the rather depleted chemical arsenal of nucleic acids, and the inclusion of modified nucleoside triphosphates (dN*TPs) in selection experiments could represent a means to bypass this shortcoming (*vide infra*) [135,136].

**Figure 6:**
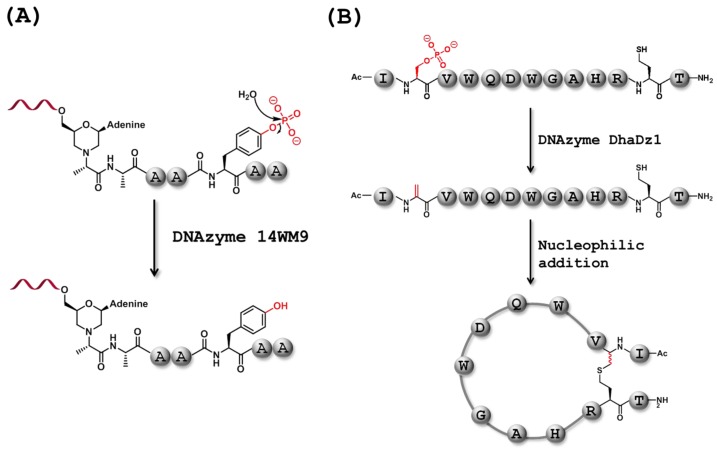
Reactions catalyzed by P-O and C-O bond breaking DNAzymes. (**A**) DNAzyme 14WM9 catalyzes the hydrolysis of phosphomonoesters on amino acid side-chains [137]; (**B**) DNAzyme DhaDz1 catalyzes the elimination reaction of a phosphate group of a serine side-chain [139].

The research group led by Silverman also selected a DNAzyme, Dz 14WM9 (Figure 6A), capable of hydrolyzing the monophosphoester bond located on the side-chain of a tyrosine or serine, themselves embedded in a hexameric peptide substrate [137]. This phosphatase mimic catalyzed the Zn^2+^-dependent dephosphorylation under both single-turnover (*k*_obs_ = 0.19 min^−1^ on tyrosine and 5.2 × 10^−3^ min^−1^ on serine) and multiple-turnover (6 turnovers in 24 h, 15 turnovers in 96 h with an unbound hexapeptide substrate containing a phosphorylated tyrosine) reaction conditions.

Cyclic peptides are alluring targets in drug discovery due to their increased protease resistance and higher biological activities compared to their linear counterparts. On the other hand, cyclic peptides also represent more challenging synthons due to the favored *E* conformation of amide bonds which impedes the cyclization of small to medium-sized peptides and the occurrence of intermolecular reactions that compete with macrocyclization in larger systems [138]. In this context, a selection experiment was devised to evolve DNAzymes capable of eliminating a phosphate group on the side-chain of serine. After nine rounds of selection using the same hexapeptide substrate containing a phosphorylated serine as used in the selection of Dz 14WM9, DNAzyme DhaDz1 was isolated [139]. This DNAzyme catalyzed the elimination reaction with an appreciable rate constant (*k*_obs_ = 4.7 × 10^−3^ min^−1^) in the presence of three metal cofactors (Zn^2+^, Mn^2+^, and Mg^2+^) under single-turnover conditions. Importantly, DhaDz1 was capable of multiple-turnover (6–7 turnovers in 96 h) with an unbound hexapeptide substrate. DhaDz1 was also used to synthesize an analog of the cyclic peptide compstatin, a highly selective inhibitor of protein-protein interactions (Figure 6B). Briefly, the linear peptide precursor containing a phosphorylated serine and a homocysteine, was subjected to the DhaDz1-mediated elimination reaction (under single-turnover conditions) which led to the alkene precursor that readily underwent base-induced alkylation by the thiol functionality.

## 3. DNAzymes Catalyzing Bond Formation

DNAzymes are emerging members in the field of functional nucleic acids and since the advent of the first Pb^2+^-dependent RNA cleaving DNA catalysts, most research efforts were dedicated to the isolation of (RNA) bond-cleaving species due to their potential therapeutic and diagnostic appeal and the relative ease of selection. This might explain why only a rather restricted number of bond-forming DNAzymes have been evolved, especially when compared to their RNA surrogates. Indeed, ribozymes have been evolved to catalyze a variety of bond-forming reactions including C-C bonds [140,141], aminoacyl-RNA linkages [142,143,144], and peptide bonds [145,146,147]. Despite this comparatively lower abundance, various bond-forming DNAzymes have been reported and will be highlighted in this section.

### 3.1. C-C Bond Forming DNAzymes

**Figure 7:**
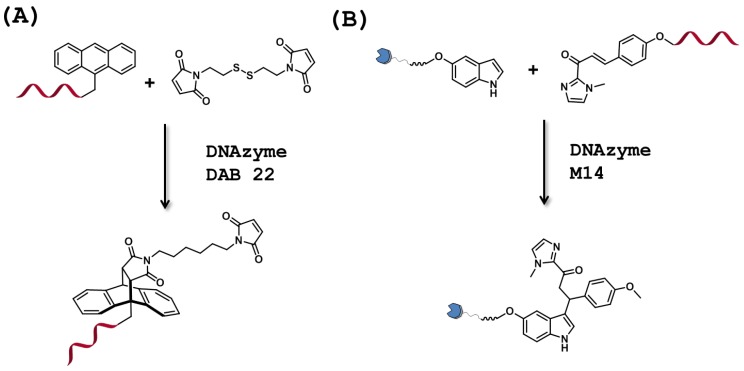
Reactions catalyzed by C-C bond-forming DNAzymes. (**A**) DNAzyme DAB 22 catalyzes the Diels-Alder reaction [149]; (**B**) DNAzyme M14 catalyzes the Friedel-Crafts alkylation reaction [150].

The Diels-Alder reaction is an important and versatile reaction in organic chemistry that enables the formation of carbon-carbon bonds. The important structural reorganization occurring in the transition state of this [4+2] cycloaddition is thought to be central for the recognition by catalytic biomolecules [19]. For instance, the first ribozyme with Diels-Alderase activity was evolved by including a UTP equipped with a methylpyridyl unit in the selection experiment [140]. The resulting ribozyme catalyzed the reaction between an acyclic diene and a maleimide-based dienophile, albeit with a modest efficiency (*k*_cat_/*K*_m_ = 237 M^−1^·min^−1^). A crystallographic structural investigation revealed that the ribozyme formed a hydrophobic pocket which dictates the stereoselective outcome of the reaction as well as the binding of the reaction components [148]. After this initial discovery, a more proficient and unmodified ribozyme was reported by the laboratory of Jäschke [141]. The resulting ribozyme, 39M49, catalyzed the Diels-Alder reaction of maleimide dienophile and an anthracene diene (see Figure 7A) with an appreciable efficiency (*k*_cat_/*K*_m_ = 10^4^ M^−1^·min^−1^). With the isolation of efficient ribozymes for the Diels-Alder reaction, arose the question of whether DNA could equally promote this cycloaddition. In order to address this interesting point, Silverman *et al.* carried out selection experiments using oligonucleotide pools that were fully randomized or that bore some sequence similarity to ribozyme 39M49 (*i.e.*, the 36 nucleotides constituting the randomized region had 70% probability of being identical to 39M49) [149]. Both oligonucleotide libraries were asked to catalyze the same Diels-Alder reaction as 39M49, with the difference that the maleimide reaction partner was connected to a second maleimide unit in order to trap the ensuing products rather than using a biotin moiety as in the ribozyme selection protocol (Figure 7A) [141,149]. Robust catalytic activities were observed in both selection experiments, and one particular clone (DAB 22) resulting from the biased library was sequenced. While the exact Michaelis-Menten parameters could not be obtained for DAB 22, the apparent second-order rate constant (*k*_app_) for the in *trans* reaction compared favorably to that of ribozyme 39M49 (0.7 M^−1^·s^−1^ *vs.* 0.8 M^−1^·s^−1^, respectively), suggesting that both DNA and RNA could catalyze this cycloaddition with similar efficiency.

The Friedel-Crafts alkylation is another synthetically relevant method for the formation of carbon-carbon bonds. Although often carried out under strictly anhydrous conditions, Friedel-Crafts reactions in water have been reported [151]. Interestingly, a hybrid catalyst consisting of a complex of 4,4′-dimethyl-2,2′-bipyridine-Cu^2+^ and salmon testes DNA was recently shown to be capable of promoting an asymmetric Friedel-Crafts alkylation in aqueous medium with high yields and enantioselectivities [152]. DNAzymes represent a valuable alternative for the catalysis of Friedel-Crafts alkylations due to their single-stranded nature and their catalytic promiscuity. In this context, McNaughton *et al.* set out to identify a DNAzyme that could catalyze this C-C bond forming reaction [150]. In the selection setup, an indole moiety was connected to a biotin residue which served in the isolation of the catalytically active species (gel-shift), while the other reaction partner, an acyl imidazole, was attached to the primer which was used in PCR to generate the randomized library (Figure 7B). Application of seven rounds of *in vitro* selection in the presence of Cu^2+^ led to the isolation of DNAzyme M14 that catalyzed the Friedel-Crafts reaction both in *cis* and in *trans* and with appreciable product yields (over 70% in 24 h). Further bond forming DNAzymes, for instance catalyzing the Michael-addition or the aldol reactions, will certainly be reported in the future since precedents exists for RNA [153,154].

### 3.2. Ligation Reactions and Modification of Peptide Substrates

**Figure 8:**
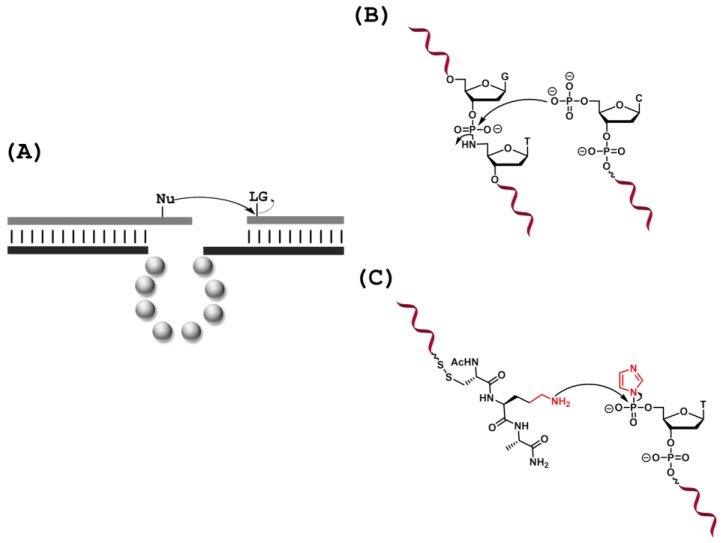
(**A**) General representation of DNAzyme catalyzed ligation reactions. A nucleophile (e.g., an RNA 2′-OH, a terminal 3′-OH, hydroxyl groups of serine and tyrosine, or an amine) located on a substrate attacks an electrophilic position (e.g., 5′-triphosphate, 5′-phosphorimidazolide) located on the second substrate with the concomitant eviction of a leaving group; (**B**) Catalyzed ligation reaction by hydrolysis of a phosphoramidate bond [162]; (**C**) DNAzyme catalyzed nucleophilic attack of an amine group of a lysine residue on a 5′-phosphorimidazolide unit [172].

DNA-mediated catalysis of ligation reactions, *i.e.*, the attack of a nucleophilic group located on one substrate on an electrophilic site positioned on another (Figure 8A), is an alluring method for the synthesis of various biopolymers [155]. Initial efforts mainly focused on DNA ligation, and shortly after the discovery of the first DNAzyme, a DNA catalyst mimicking the activity of the T4 DNA ligase was evolved [156]. The Zn^2+^/Cu^2+^-dependent DNAzyme E47 was isolated after nine rounds of selection and catalyzed the nucleophilic attack of a terminal 5′-hydroxyl group onto a phosphorimidazolide unit located at the 3′-end of a second DNA substrate, thus generating a phosphodiester linkage with an appreciable rate constant (*k*_cat_ = 0.07 min^−1^). More recent selection experiments have led to the isolation of DNAzymes capable of self-phosphorylation [157,158,159], adenylation [160], and ligation [161], therefore performing all the steps required for the T4 DNA ligase-mediated splint ligation of DNA oligonucleotides [29]. A slightly different approach was employed to isolate DNAzymes capable of ligating two 3′- and 5′-phosphorylated trinucleotidic substrates to yield a hexameric DNA sequence with an internal 3′-5′ pyrophosphate linkage [162]. In this case, a scissile phosphoramidate bond was chosen as the leaving group, which could undergo a nucleophilic attack by the 5′-phosphate unit of one of the trinucleotidic substrates, in analogy to the catalytic mechanism followed by certain DNA ligases (Figure 8B). The isolated DNAzyme utilized the 5′-phosphorylated trinucleotide as a cofactor and led to a ≥10^3^-fold rate enhancement compared to the uncatalyzed reaction. In addition, a DNAzyme capable of catalyzing the synthesis of branched DNA has also been isolated: DNAzyme 8LV13 catalyzes the addition of a 2′-OH moiety of a single ribo(adenosine) nucleotide embedded in a DNA oligonucleotide on an electrophilic 5′-adenylate center with high efficiency (*k*_obs_ = 0.1 min^−1^) [163].

RNA ligation is of particular interest due to the synthetic challenges inherent to the chemical, solid-phase synthesis of longer sequences (*i.e.*, >100 nt). The Silverman lab has been particularly active in the development of RNA-ligating DNAzymes. Comprehensive reviews can be found on this topic [22,29,164], and only a few key and recent results will be highlighted herein. The first representative of RNA-ligating DNAzymes, Dz9A5, catalyzed the formation of non-native 2′-5′-RNA phosphodiester bonds (*k*_obs_ = 0.013 min^−1^) by promoting the opening of a 2′,3′-cyclic phosphate by a 5′-OH group [165]. This initial discovery was shortly followed by the isolation of DNAzyme 7S11, which specifically recognizes an unpaired adenosine nucleotide on one RNA substrate and mediates the nucleophilic attack of the 2′-hydroxyl group on the α-phosphorous atom of a 5′-triphosphate unit on the other RNA substrate (with a rate of: *k*_obs_~0.5 min^−1^), thus also generating 2′-5′ branched RNA linkages [166]. Dz 7S11 and related 2′-5′ RNA-ligating DNAzymes have been employed for the labelling of RNA molecules with a variety of functional groups [167,168] or for mechanistic investigations [169]. Interestingly, lanthanide cations (Tb^3+^ particularly), which often inhibit RNA-cleaving DNAzymes [170], have been shown to massively enhance the catalytic efficiency of RNA-ligating DNAzymes–most likely by promoting the formation of the catalytically active structures [171].

For synthetic purposes and with the aim of expanding the chemical nature of accessible substrates, catalytic nucleic acids that function in organic media would represent a valuable tool. While some small ribozymes such as the hammerhead ribozyme have been shown to accommodate reasonably well to media containing organic co-solvents [173], RNA-cleaving DNAzymes such as Dz8-17 are rather reluctant to operate under such conditions [174]. Rather serendipitously, an RNA-ligase DNAzyme was identified in a selection experiment that can operate in the presence of various organic co-solvents [175]. This behavior was then found to be rather general for RNA-ligating DNAzymes, since two other catalysts still showed appreciable ligation activity in media containing high concentrations of organic co-solvents (e.g., *k*_obs_ values of 0.1 min^−1^ were observed for media containing up to 50% EtOH).

The catalysis of the ligation of nucleic acid-based substrates is of importance for biochemical and biotechnological applications since this represents a valid alternative to splint ligations and to solid-phase synthesis. In addition, Watson-Crick base-pair interactions between oligonucleotide substrates and DNAzymes obliterate the need for allocating additional energy to binding the substrate to the enzyme. These reasons account for the intense research efforts that focused on the fabrication of catalytic nucleic acids capable of ligating oligonucleotides together. On the other hand, the inclusion of non-oligonucleotide substrates in selection experiments could broaden the scope and increase the synthetic usefulness of DNAzymes. In this context, the Silverman laboratory developed numerous DNAzymes capable of recognizing and modifying peptide substrates [136]. In a seminal contribution, DNAzymes were evolved to catalyze the nucleophilic attack of hydroxyl groups located on the side-chain of a tyrosine (DNAzyme Tyr1, *k*_obs_ = 0.06 min^−1^) unit embedded in a DNA substrate on a 5′-triphosphate unit on an RNA substrate, resulting in the formation of RNA-nucleopeptide linkages [176]. This catalytic activity was then expanded to other side-chains (e.g., serine [177] or phosphorylated tyrosine [178]), non-nucleosidic substrates (mainly consisting of tri- or hexapeptides embedded in DNA oligonucleotides) and for the phosphorylation of peptide substrates [179]. Surprisingly, DNAzymes could not compensate for the apparently lower nucleophilicity of the NH_2_ functionality of the side-chain of lysine and the 5′-triphosphate had to be replaced by a more electrophilic 5′-phosphorimidazolide (5′-Imp) unit to enable a positive outcome of the selection experiments (Figure 8C) [175]. In this context, a particular DNAzyme, 9DT105, was isolated from one of these *in vitro* selections and was shown to ligate the terminal amino group of the lysine substrate to the 5′-Imp-modified substrate with an appreciable efficiency (*k*_obs_ = 1.7 × 10^−3^ min^−1^; 50% yield).

Undoubtedly, the diversity of the nature of the substrates accepted by DNAzymes as well as their activities are both constantly increasing and the isolation of catalytic species capable of more complex operations on larger peptides or even proteins seems to be imminent.

## 4. DNAzymes Catalyzing other Reactions

**Figure 9:**
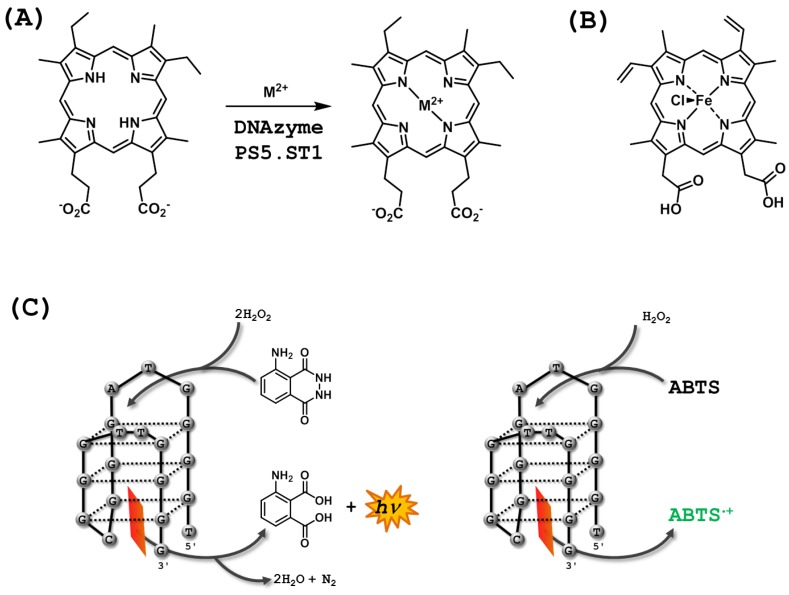
(**A**) Porphyrin metalation catalyzed by DNAzyme PS5.ST1 (M = Zn^2+^ or Cu^2+^) with mesoporphyrin IX (MPIX) as a substrate [180]; (**B**) Chemical structure of the hemin (Fe(III)-protoporphyrin IX) cofactor; (**C**) Reactions catalyzed by the DNAzyme PS2.M with hemin: reaction of luminol with hydrogen peroxide to yield the corresponding diacid and chemiluminescence [183,193]; oxidation of the chromogenic ABTS (2,2′-azino-bis(3-ethylbenzthiazoline-6-sulfonic acid)) to the corresponding radical cation ABTS^·+^ which has a distinctive green color [193].

As aforementioned, DNAzymes capable of recognizing and utilizing non-nucleosidic substrates in general, and small molecules particularly, are rather scarce. However, an important example is DNAzyme PS5.ST1 which mimics the action of chelatases by inserting metal cations (Zn^2+^ or Cu^2+^) into porphyrin scaffolds (Figure 9A) [180]. A key step in the isolation of Dz PS5.ST1 was to challenge a very long (N_228_) randomized oligonucleotide population to bind to a bead-bound, constrained porphyrin that mimicked the transition state adopted in the active site of chelatases [181]. The resulting aptamers, which bound to various porphyrin substrates with low-micromolar affinities, were then tested for their capacity at catalyzing the insertion of M^2+^ cations into mesoporphyrin IX [180]. One particular aptamer coined DzPS5.ST1, inserted Cu^2+^ into MPIX with a catalytic efficiency of 79 min^−1^·M^−1^ which represents a ~1400-fold enhancement compared to the uncatalyzed background reaction. An optimization of the sequence by rational design and improvement of the reaction conditions made it possible to identify a shortened and highly proficient (*k*_cat_/*K*_m_ = 3.3 × 10^4^ min^−1^·M^−1^) version (PS5.M) of the initial DNAzyme [182]. Following the discovery and optimization of DzPS5.ST1 as a porphyrin metalation catalyst, its function was extended to the role as a highly efficient peroxidase mimic [183]. Indeed, the metalated porphyrin hemin (Figure 9B) was found to tightly bind to DNAzyme PS5.M and by the same token to act as a potent inhibitor of the metalation reaction. On the other hand, when hemin was complexed to DzPS2.M—another aptamer isolated in the initial selection experiment (*vide supra*)—in the presence of a detergent (to avoid aggregation and μ-oxo dimer formation) and hydrogen peroxide, ABTS was oxidized to the corresponding radical cation ABTS^·+^ (Figure 9C) with a rate constant ~250-fold superior to that of the hemin-mediated background reaction. Similarly to peroxidases such as the horseradish peroxidase, DzPS2.M can oxidize a variety of substrates including luminol (Figure 9C) [184] or NADH [185] which then serve as indicators for the progress of the reaction. Due to these favorable properties, this peroxidase mimic has found numerous applications essentially in the field of biosensing, DNA detection, and structural investigations [186,187,188,189]. Interestingly, a variant of DzPS2.M was conjugated with polyethylene glycol (PEG) units and shown to maintain the peroxidase activity in methanol ‒representing the first example of a DNAzyme capable of working in completely organic media [173]. More recently, DNAzyme PS2.M was appended at the C5 position of the nucleobase of a dUTP and incorporated into DNA by primer extension reactions mediated by the *KF exo*^−^ DNA polymerase [190]. This ingenious system was then employed for the naked eye detection of point mutations, particularly the T1796A substitution in the B type Raf kinase gene.

As for most DNAzymes, the exact mechanism has not been fully elucidated so far. However, it is believed that the G-quadruplex nature of the DNAzyme offers a hydrophobic binding site for the hemin cofactor [183,191]. Following binding of the hemin inside of the G-quadruplex region, the axial chlorine is displaced by an H_2_O_2_ molecule which is then cleaved in a process presumably mediated by a guanine nucleotide [188]. Loss of a water molecule then leads to the oxidation of the iron(III) prosthetic group to the highly reactive, hemin-bound radical cation porph:Fe(IV)=O^·+^, which in turn withdraws one electron from the ABTS substrate to form both the ABTS^·+^ product and a more stable intermediate, ferryl-hemin (porph:Fe(IV)=O) [188,192]. After this one electron transfer, the ferryl-hemin moiety abstracts another electron from a second H_2_O_2_ molecule, which then leads to the formation of a ferric-superoxy (porph:Fe(III)-O_2_^·−^) intermediate. Finally, interaction of this ferric-superoxy species with a second ABTS substrate molecule eventually restores the initial hemin:iron(III) complex [188].

## 5. Toward an Expansion of the Catalytic Repertoire of DNAzymes

**Figure 10:**
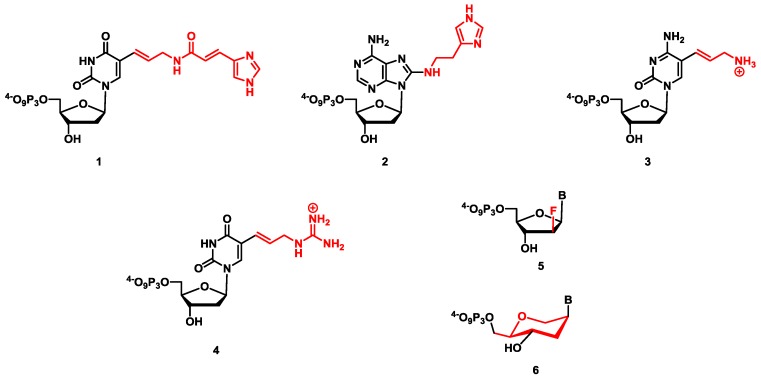
Chemical structures of modified nucleoside triphosphates (dN*TPs) used in *in vitro* selection experiments for the generation of modified DNAzymes: C5-imidazole-functionalized dUTP (**1**), dA^im^TP (**2**), dC^aa^TP (**3**), dU^ga^TP (**4**), FANA-NTPs (**5**), and HNA-NTPs (**6**). The chemical modifications are highlighted in red.

Most known DNAzymes require one or multiple cofactors (usually divalent metal cations) for optimal activity, and this often at concentrations that exceed those found in cells. A further caveat is that DNAzymes, like all unmodified functional nucleic acids, are prone to nuclease degradation, which is clearly detrimental for certain (*in vivo*) applications. In addition, some reactions such as amide bond cleavage seem to be inaccessible to catalytic nucleic acids, probably due to the lack of suitable functional groups [135,136,194]. Thus, in order to mitigate these drawbacks, DNAzymes can be equipped with non-natural functional groups (Figure 10), either during or after the selection process [78,195,196]. The inclusion of modifications directly into the selection process via the co-polymerization of dN*TPs [197,198] offers the advantages of providing (i) optimized catalytic systems that do not need much further optimization; (ii) an additional dimension—*i.e.*, chemical space—that can be explored during selection experiments; (iii) a facile modulation of the nature of the functional groups involved. This strategy was first successfully applied to generate modified aptamers with enhanced properties and was later hijacked for the generation of DNAzymes [14,15].

**Figure 11:**
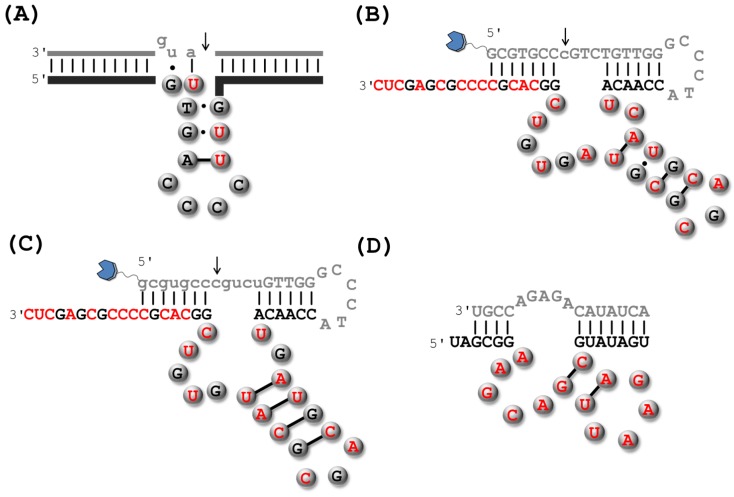
Sequences and hypothetical 2D structures of DNAzymes obtained with dN*TPs included in the *in vitro* selections: Dz16.2-11 (**A**), Dz9-86 (**B**), Dz12-91 (**C**), and FANAzyme FR17_6 (**D**). The bold red letters highlight the position of the modifications while arrows indicate the cleavage sites.

The first reported DNAzyme obtained by the inclusion of a dN*TP in the *in vitro* selection process was Dz16.2-11 (Figure 11A) [199]. This Zn^2+^-dependent RNA-cleaving DNAzyme contains modified dUMP (**1**) units (Figure 10) and hydrolyzes all-RNA substrates with a very high catalytic efficiency (*k*_cat_/*K*_m_~10^8^ min^−1^·M^−1^), presumably via a mechanism reminiscent of enzymes such as carboxypeptidase A.

Paralleling the discovery of Dz16.2-11, other selection experiments involving up to three dN*TPs used *in lieu* of their natural counterparts focused on trying to obliterate the need for M^2+^ cofactors for RNA-cleavage [68,200,201,202,203,204]. A first example was reported by Perrin *et al.* who used the combination of triphosphates bearing histidine- (dA^im^TP (**2**)) and lysine-like (C5-allyamino-dUTP, dU^aa^TP) side-chains to generate a ribonuclease mimic promoting the M^2+^-independent cleavage of RNA substrates with high efficiency (*k*_cat_/*K*_m_ = 5 × 10^5^ min^−1^·M^−1^) [205,206]. In both examples, the imidazole units play a fundamental role, either as chelators of Zn^2+^ or in participating in general acid/general base catalysis. Additionally, the pK_a_ of imidazole residues appended on nucleobases has recently been shown to be variable depending on the nature of the interaction with the DNA context [207].

With the intent of further increasing the catalytic efficiency and broadening the substrate scope of M^2+^-independent DNAzymes, a third, guanidinium-modified dN*TP was included in selection experiments. Indeed, modified oligonucleotide populations obtained by the co-polymerization of the dN*TPs (**2**), (**3**), and (**4**) were challenged to cleave substrates containing either a single embedded scissile ribo(cytosine)phosphodiester linkage (Figure 11B) or a stretch of RNA nucleotides (Figure 11C) in the exclusion of M^2+^. The resulting DNAzymes, Dz9-86 [202] and Dz12-91 [204], respectively, could cleave their respective substrates with high first-order rate constants (*k*_obs_ = 0.13 min^−1^ and 0.06 min^−1^) at 37 °C in the absence of metal cofactors. Surprisingly, both DNAzymes are very similar both in terms of sequence composition and topology despite having been selected for rather different substrates. Thus, it appears that minute alterations in the catalytic core account for the higher catalytic efficiency of Dz12-91 compared to Dz9-86. Indeed, the rate constants for self-cleavage observed for Dz12-91 were higher than those for Dz9-86 regardless of the nature of the substrate (0.13 *vs.* 0.0014 min^−1^ for all-RNA and *k*_obs_ = 0.27 min^−1^ *vs.* 0.13 min^−1^ for the substrate with the single ribo(cytosine) nucleotide, respectively).

More recently, engineered polymerases [208] that enable the replication of xeno-nucleic acids XNAs with sugar-altered structures were used in selection experiments to generate XNAzymes [209,210]. Four types of XNA chemistries, including FANAs (**5**) and HNAs (**6**), were used to fabricate all-RNA cleaving XNAzymes [210]. The most efficient catalyst, FANAzyme FR17_6 (Figure 11D), hydrolyzed an all-RNA substrate with an appreciable rate constant and with multiple turnover (*k*_obs_ = 0.026 min^−1^; 35 turnovers in 96 h). Interestingly, while all these XNAzymes were novel sequences, some topological similarities with the DNAzymes 8-17 and 10-23 were observed, suggesting that each selection scheme might have a limited subset of answers to the particular reaction it is challenged to undertake. Furthermore, XNAzymes capable of ligating two RNA substrates were obtained by application of a selection protocol devised for unmodified DNA (Figure 8A). Even though the most efficient XNA ligase (based on FANA chemistry) catalyzed the ligation of the two substrates with a rather low rate constant (*k*_obs_ = 2 × 10^−4^ min^−1^), the rate enhancement compared to the uncatalyzed reaction is substantial (~10^4^). Finally, a FANAzyme could also be selected to ligate two FANA substrates (*k*_obs_ = 0.04 min^−1^) and the synthetic usefulness of this XNAzyme was underscored by generating a 100 nt long FANA oligonucleotide.

A large variety of dN*TPs, equipped with amino acid-like residues as well as non-natural functional groups, have been developed for their use in *in vitro* selection experiments to generate DNAzymes with an expanded catalytic repertoire [135,136,198,211,212,213,214,215,216]. However, no further modified DNAzymes have been reported so far.

## 6. Conclusions and Outlook

Since their recent advent, DNAzymes have grown into a very potent type of functional nucleic acids with ramifications into multiple applications including therapeutics, biosensing, DNA nanotechnology, and organic synthesis. The over 20 different chemical transformations catalyzed by DNAzymes summarized herein are a testimony for the growing importance of these biocatalysts. Even though DNAzymes are still rather weak rivals for their proteinaceous and to a certain extent their RNA counterparts [217], progress made in the development of these DNA-based catalysts cannot be overlooked: diffusion-controlled catalytic efficiencies have been achieved for RNA-cleaving DNAzymes under specific (high [M^2+^]) conditions and DNAzymes have recently been evaluated in clinical trials for the treatment of skin cancer and asthma. However, despite numerous favorable assets, DNAzymes are not quite ready for a general use in practical applications and some challenges need to be overcome: firstly, the catalytic efficiencies (*k*_cat_/*K*_m_ values) regularly lie below the standards required for *in vivo* applications. Secondly, the strong M^2+^-dependence for optimal catalytic activity might also result in a predicament for cellular assays since the concentrations of free M^2+^ in cells are usually well below the levels required by the DNAzymes (e.g., free [Zn^2+^] and [Mn^2+^] lie in the high picomolar [218] and low micromolar [219] range, respectively). Thirdly, mechanistic and structural studies are required to gain a better understanding of the mode of action of DNAzymes and possibly enable their rational design. Lastly, some potentially useful reactions (e.g., amide bond cleavage, Michael-addition reaction, organocatalysis…) still elude DNAzymes and both the selectivity and recognition of non-oligonucleotide substrates such as peptides and small organic compounds still need to be improved. In this context, the combination of Darwinian evolution, the robustness of the DNA scaffold, and the possible inclusion of a broad array of additional functional groups will be pivotal to circumvent these limitations, while the development of new selection strategies will also greatly contribute to facilitate the selection process leading to the fabrication of DNAzymes [220].

Taken together, DNAzymes represent a very valuable addition to the armamentarium of existing biocatalysts and have made an important step out of infancy. Their chemical repertoire is continuously increased as well as their efficiencies. Progress in selection strategies will certainly help in the development of DNAzymes with hitherto unknown reactivity for their further use in practical applications. Particularly, future efforts should be invested in the development of DNAzymes capable of selectively recognizing proteins and carbohydrates for their further processing since they would represent invaluable biological and synthetic tools.

## Abbreviations

*cis*/*trans* catalyst: intra-/inter- molecular catalyst; dinucleotide junction: bases immediately flanking the scissile bond; dN*TPs: modified nucleoside triphosphates; Dz: DNAzyme, deoxyribozyme, or DNA enzyme; nt: nucleotide; SELEX: systematic evolution of ligands by exponential enrichment; ssDNA or ssRNA: single-stranded DNA or RNA, respectively; 5′-Imp: 5′-phosphorimidazolide; FANA: 2′-deoxy-2′-fluoro-β-d-arabinonucleic acid; XNA: xeno-nucleic acids.
